# Oligomeric Proanthocyanidins Confer Cold Tolerance in Rice through Maintaining Energy Homeostasis

**DOI:** 10.3390/antiox12010079

**Published:** 2022-12-29

**Authors:** Juncai Li, Baohua Feng, Pinghui Yu, Weimeng Fu, Wenting Wang, Jie Lin, Yebo Qin, Hubo Li, Tingting Chen, Chunmei Xu, Longxing Tao, Zhihai Wu, Guanfu Fu

**Affiliations:** 1 Agronomy College, Jilin Agricultural University, Changchun 130118, China; 2 National Key Laboratory of Rice Biology, China National Rice Research Institute, Hangzhou 310006, China; 3 Zhejiang Agricultural Technology Extension Center, Hangzhou 310020, China

**Keywords:** antioxidant capacity, ATPase, cold tolerance, energy homeostasis, oligomeric proanthocyanidins, *Oryza sativa* L.

## Abstract

Oligomeric proanthocyanidins (OPCs) are abundant polyphenols found in foods and botanicals that benefit human health, but our understanding of the functions of OPCs in rice plants is limited, particularly under cold stress. Two rice genotypes, named Zhongzao39 (ZZ39) and its recombinant inbred line RIL82, were subjected to cold stress. More damage was caused to RIL82 by cold stress than to ZZ39 plants. Transcriptome analysis suggested that OPCs were involved in regulating cold tolerance in the two genotypes. A greater increase in OPCs content was detected in ZZ39 than in RIL82 plants under cold stress compared to their respective controls. Exogenous OPCs alleviated cold damage of rice plants by increasing antioxidant capacity. ATPase activity was higher and poly (ADP-ribose) polymerase (PARP) activity was lower under cold stress in ZZ39 than in RIL82 plants. Importantly, improvements in cold tolerance were observed in plants treated with the OPCs and 3-aminobenzamide (PARP inhibitor, 3ab) combination compared to the seedling plants treated with H_2_O, OPCs, or 3ab alone. Therefore, OPCs increased ATPase activity and inhibited PARP activity to provide sufficient energy for rice seedling plants to develop antioxidant capacity against cold stress.

## 1. Introduction

Rice is one of the most important crops in East and Southeast Asia [1]. As a subtropical or tropical crop, rice is susceptible to cold stress during the seedling and reproductive stages. Cold stress that occurs at the seedling stage leads to chlorosis of leaves, a reduction in the number of tillers, damage to the root system, and death [2,3,4]. This damage significantly decreases the yield and quality of the rice and restricts the growth of planting area of early rice in China [5,6,7,8].

Cold stress inhibits normal active oxygen metabolism in plant and causes changes to the cell membrane structure, enzyme functions, osmotic substances, and stomatal conductance [9,10,11,12]. These changes can damage or kill plants, which show symptoms such as wilting, slow growth, yellowing, and local tissue necrosis [13,14,15,16,17]. Notably, some plants grow normally under cold stress, which is related to cold tolerance and cold acclimation. The acquisition of cold acclimation and cold tolerance is a complex process, and a large number of genes, as well as changes in antioxidant capacity and membrane structure, are involved in this process [18,19,20,21]. Among them, oxidative stress affects plant growth and development; thus, enhancing antioxidant capacity by salicylic acid, quercetin, melatonin, and abscisic acid is important for rice seedlings to survive in abiotic stress including cold stress [22,23,24].

Flavonoids including proanthocyanidins (PAs) are a class of polyphenols characterized by three six-member rings with two aromatic rings linked by an oxygen-containing heterocycle. The flavonoids play a multitude of roles in plants, one of which functions as strong non-enzymatic antioxidants to effectively remove excess reactive oxygen species (ROS) caused by abiotic stressors, such as cold [25,26,27]. Thus, the metabolism of flavonoid has attracted considerable interest in plants under abiotic stress [28]. PAs are oligomers or polymers of flavan-3-ol units and are the final product of the flavonoid biosynthetic pathway, which are prominent flavonoid compounds in seed coats, leaves, fruits, flowers, and bark [29]. The PAs are composed of different amounts of catechins (catechin) or epicatechin (epicatechin), in which the simplest proanthocyanidins are catechins, epicatechin, or dimers formed by catechins and epicatechin, as well as trimers, tetramers and even decamers [30]. According to the degree of polymerization, dipentamers are usually called oligomeric proanthocyanidins (OPC), and those above pentamers are called high polymerized proanthocyanidins (PPC) [30]. It has been reported that the oxidative resistance could be enhanced by accumulating OPCs in rose [31]. Similarly, tea plants accumulate OPCs to enhance antioxidant capacity under drought stress [32]. OPCs are also involved in the plant’s defense against high radiation and cold stress [33,34,35,36]. These results indicated that OPCs could improve the performance of plants under abiotic stress.

As well-known, OPCs act as an important antioxidant to alleviate oxidative damages on plants caused by abiotic stresses [25,26,27], whereas how it strengthen antioxidant capacity which is a high energy cost process remains ambiguity. Energy status is an important factor involved in regulating plant growth and development irrespectively of under normal or abiotic stress conditions [37,38,39]. The activation of poly (ADP-ribose) polymerase (PARP) as well as inhibition of ATPase are always presented in plants under abiotic stress including cold stress [37,39], which cause energy disorders in plants, aggravate energy deficiencies, and impair antioxidant capacity, and thus result in stagnant growth or death [40,41,42]. In this process, plenty of NAD^+^ are consumed in plants when the PARP is activated by abiotic stress [43,44]. In contrast, plants treated with 3-Aminobenzamide (PARP inhibitor, 3ab) conferred resistance to oxidant stress through reducing NAD^+^ consumption and maintaining energy homeostasis [39,45]. Further, the ATPase could be activated to enhance the abiotic resistance in plants [46,47,48]. These results indicate that energy balance plays an important role in the plants’ response to abiotic stress. As reported, lower accumulation of PAs were showed in the Arabidopsis thaliana mutant transparent testa 13 (tt13) caused by disruption of the gene encoding the P3A -ATPase AHA10 [49]. This gene functioned as a proton pump in the tonoplast of seed coat endothelium cells, and generated the driving force for TT12-mediated transport of PA precursors to the vacuole [49]. This indicated that the formation of PAs in the seed coat endothelium of Arabidopsis thaliana required the participation of plasma membrane H+-ATPase [50]. Though such results have not been found in rice [51], the plasma membrane H+-ATPase play key roles in rice growth and development through enhancing nutrient uptake and photosynthesis as well as the removal of ROS [52,53,54]**.** Therefore, we speculate that the increase in OPCs and antioxidant capacity under cold stress may be related to energy metabolism. In this study, two rice genotypes with different cold tolerances were subjected to cold stress at the seedling stage. The antioxidant capacity, OPCs, carbohydrates, energy metabolism, maximum fluorescence quantum efficiency (Fv/Fm), and relative electrical conductance (REC) were measured to reveal the mechanism underlying how OPCs affect energy status to confer cold resistance in rice seedling plants.

## 2. Materials and Methods

### 2.1. Plant Materials and Growth Conditions

Two rice genotypes, such as Zhongzao 39 (ZZ39) and its recombinant inbred line RIL82, were selected for study. The seeds were soaked for 48 h, germinated at 37 °C for 24 h, and directly sown in a pot (height 10 cm, diameter 10 cm) in an artificial climate chamber. The temperature was controlled at 28/22 °C (day/night), with a light intensity of 1000 μmolm^−2^s^−1^ and relative humidity of 70%. Rice seedlings with three leaves were thinned to five per pot. All rice seedlings were divided into two groups; one group was subjected to a 24 h cold stress treatment with a stress temperature of 13/10 °C (day/night) and the other group served as a control treatment with a control temperature of 28/22 °C. During this period, the relative humidity was maintained at 70%, and light intensity was maintained at 300 µmolm^−2^s^−1^. After 24 h of cold stress, the Fv/Fm of the first fully expanded leaves were determined, and then these leaves were sampled to measure or analyze the REC, OPCs content, and transcriptome. Finally, the mortality rate of the seedling plants caused by cold stress was investigated.

### 2.2. Exogenous Spraying with Different Concentrations of OPCs under Cold Stress

Five OPCs concentrations (Shanghai Ruiyong Biotechnology Co., Ltd., Shanghai, China) including 0, 0.05%, 0.1%, 0.2% and 0.4% (m/v) were prepared. About 1 h before the cold stress, 10 mL OPCs solutions containing 0.1% (*v*/*v*) Tween 20 (Sinopharm Group Chemical Reagent Co., Ltd., Shanghai, China) as a surfactant were exogenously sprayed on the rice seedling plants per pot. After the 24 h cold stress treatment, the first fully expanded leaf was sampled to determine the REC, malondialdehyde (MDA), hydrogen peroxide (H_2_O_2_), antioxidant enzyme, carbohydrate, ATP, ATPase, and poly (ADP-ribose) polymerase (PARP) levels.

### 2.3. Exogenous Spraying of OPC + 3-Aminobenzamide (PARP Inhibitor, 3ab) Combination under Cold Stress

A solution containing 0.1% (m/v) OPCs, 1 mM 3ab solution (Shanghai Saan Chemical Technology Co., Ltd., Shanghai, China), and 0.1% (*v*/*v*) Tween20 was sprayed onto the rice seedling plants 1 h before the cold stress. After 24 h of cold stress, the first fully expanded leaf was selected to determine the Fv/Fm, and then these leaves were sampled to analyze the H_2_O_2_, ATP, ATPase, and PARP contents.

### 2.4. Measurement of REC and Fv/Fm

According to the method of Xiong et al. [55], 0.5 g fresh leaves were collected at the end of the cold stress, cut into about 25-mm^2^ diameter discs with a puncher avoiding the veins, and immediately immersed in a test tube containing 10 mL of deionized water for 24 h at 25 °C. After the incubation, a conductivity meter (DDA-11A; Shanghai Hongyi Instrument Co., Ltd., Shanghai, China) was used to measure the electrical conductivity (EC1) of the solution. After the sample was placed in a water bath at 80 °C for 2 h, it was cooled to 25 °C, and the electrical conductivity (EC2) was measured again. Ion leakage was calculated as the ratio between EC1 and EC2.

After the seedlings completed a 30 min dark adaptation period, the Fv/Fm values of the leaves were measured using a portable chlorophyll fluorescence spectrometer (PAM-2500 chlorophyll fluorescence system; Heinz Walz, Effeltrich, Germany) [56].

### 2.5. RNA Sequencing (RNA-seq) and Bioinformatics Analysis

The first fully expanded leaves of seedlings plants grown under control and cold temperature conditions were harvested. Total RNA was extracted from rice leaves using Trizol reagent (Shanghai Thermo Fisher Scientific Co., Ltd., Shanghai, China). The Nanodrop ND-2000 spectrophotometer (Thermo Scientific, Waltham, MA, USA) and Agilent Bioanalyzer 4150 (Agilent Technologies, Santa Clara, CA, USA) were used to detect RNA quality and concentration, respectively. The mRNA was purified with oligo (dT) magnetic beads, and the mRNA was fragmented in ABclonal First Strand Synthesis Reaction Buffer. Random primers and reverse transcriptase were used to synthesize the first-strand cDNA. The synthesized cDNA was amplified by polymerase chain reaction (PCR) and sequenced using the Illumina Novaseq 6000/MGISEQ-T7 sequencing platform. Transcriptome sequencing and analytical services were completed by Shanghai Zhongke New Life Biotechnology Co., Ltd. (Shanghai, China).

### 2.6. Quantitative Real-Time Polymerase Chain Reaction (qRT-PCR) Analysis

Total RNA was extracted and purified with the TRIPure reagent (Aidlab Biotechnologies, Beijing, China). RNA was reverse transcribed into single-stranded cDNA using the ReverTra Ace qPCR RT Master Mix (TOYOBO, Shanghai, China). The cDNA was used as the template for PCR amplification. SYBR Green I (TOYOBO) was used as the fluorescent dye, and the Thermal Cycler Dice Real-Time System II (TaKaRa Biotechnology, Dalian, China) was used for real-time fluorescent qPCR analysis. The primers were designed using PRIMER5 software and are listed in Supplementary Table S1. QRT-PCR was performed according to the method of Feng et al. [57], and the relative expression levels of the genes were analyzed by the 2^−ΔΔCT^ method.

### 2.7. OPC Contents Measurement

Based on the method of Mitsunaga et al. [58], with slight modifications, about 0.1 g of fresh leaves were placed in a 2 mL solution containing 60% ethanol and ground into a homogenate. The homogenate was shaken and extracted at 60 °C for 2 h and then centrifuged at 10,000× *g* for 10 min. Vanillin hydrochloride solution was added to the supernatant, mixed well, incubated in a water bath at 30 °C for 30 min, and absorbance was measured at 500 nm using a spectrophotometer (Lambda 25; Perkin Elmer, Freemont, CA, USA).

### 2.8. H_2_O_2_ and Lipid Peroxidation Measurements

H_2_O_2_ content was determined based on the method of Brennan and Frenkel, [59] with slight modifications. Four mL of 10 mM 3-amino-1,2,4-triazole (Bio Basic Inc, Toronto, Canada) and 0.2 g of fresh leaves were ground into a homogenate and centrifuged at 6000× *g* for 25 min. Two mL of the supernatant was added to 1 mL of 0.1% titanium tetrachloride (Shanghai Lingfeng Chemical Reagent Co., Ltd., Shanghai, China) solution containing 20% H_2_SO_4_, mixed, centrifuged, and the absorbance of the supernatant was measured with a spectrophotometer at 410 nm.

Two mL of 5% trichloroacetic acid was added to 0.1 g of fresh leaves to form a homogenate. The concentration of thiobarbituric acid (Sinopharm Group Chemical Reagent Co., Ltd., Shanghai, China) reactive substances was determined to estimate MDA content [60].

### 2.9. Antioxidant Enzyme Activity Measurements

Peroxidase (POD) activity was measured by the method of Maehly and Chance, [61]. Superoxide dismutase (SOD) activity was determined by the method of Giannopolitis and Ries, [62]. Catalase (CAT) activity was determined using the method of Zhang et al. [63].

### 2.10. Carbohydrate Measurements

Soluble sugar and starch contents were determined by the sulfuric acid-anthrone colorimetry method [64]. About 0.2 g of fresh leaves were immersed in 10 mL of absolute ethanol, heated at 80 °C for 30 min, extracted three times, the supernatant was removed to a constant volume, and activated carbon was added and shaken for 1 h to decolorize. The decolorizing solution was used to determine soluble sugar contents, and the filter residue was used to extract starch. The total non-structural carbohydrate (NSC) content was the sum of soluble sugar and starch contents.

### 2.11. ATP Content

ATP content was determined using an ATP analysis kit (Shanghai Enzyme-Linked Biotechnology Co., Ltd., Shanghai, China). About 0.1 g of fresh leaves were mixed with 1 mL of 0.1 M pH 7.4 PBS. The mixture was fully ground in an ice bath and centrifuged at 3000× *g* for 20 min and the supernatant was collected. The supernatant, standard and horseradish peroxidase (HRP)-labeled detection antibody were sequentially added to the microwells coated with the ATP capture antibody, incubated at 37 °C for 60 min, and washed thoroughly. The color is developed with the substrate 3,3′,5,5′-tetramethylbenzidine (TMB). TMB is converted into blue under the catalysis of HRP and into yellow under the action of acid. Absorbance was measured at a wavelength of 450 nm with a microplate reader (Shanghai Thermo Fisher Instrument Co., Ltd., Shanghai, China).

### 2.12. Total ATPase and PARP Content 

ATPase content and PARP content were determined using an enzyme-linked immunosorbent assay (ELISA) kit according to the manufacturer’s instructions (Shanghai Enzyme Link Biotechnology Co., Ltd., Shanghai, China). The kit was used to quantitatively detect the content of total ATPase and PARP in plant tissue samples in vitro. About 0.1 g of fresh leaves were extracted with 0.1 M pH 7.4 PBS and then centrifuged at 3000× *g* for 20 min at 4 °C, and the supernatant was collected. The supernatant, standard, and HRP-labeled detection antibody were added to the microwells pre-coated with ATPase and PARP-captured antibody in sequence, incubated at 37 °C for 60 min, and washed. The color was developed with the substrate TMB. TMB was converted into blue under the catalysis of HRP and into yellow under the action of acid. The shade of color is positively correlated with the ATPase and PARP content in the sample. Absorbance was measured at a wavelength of 450 nm with a microplate reader.

### 2.13. Statistical Analysis

Data were processed using SPSS 11.5 software (IBM Corp., Armonk, NY, USA) to detect differences. The mean values and standard errors represent data from three independent experiments. The *t*-test and two-factor analysis of variance (temperature and treatment) were used to compare the differences with the LSD test A. *p*-value < 0.05 was considered significant.

## 3. Results

### 3.1. The Responses of the Rice Seedlings to Cold Stress

**Figure 1:**
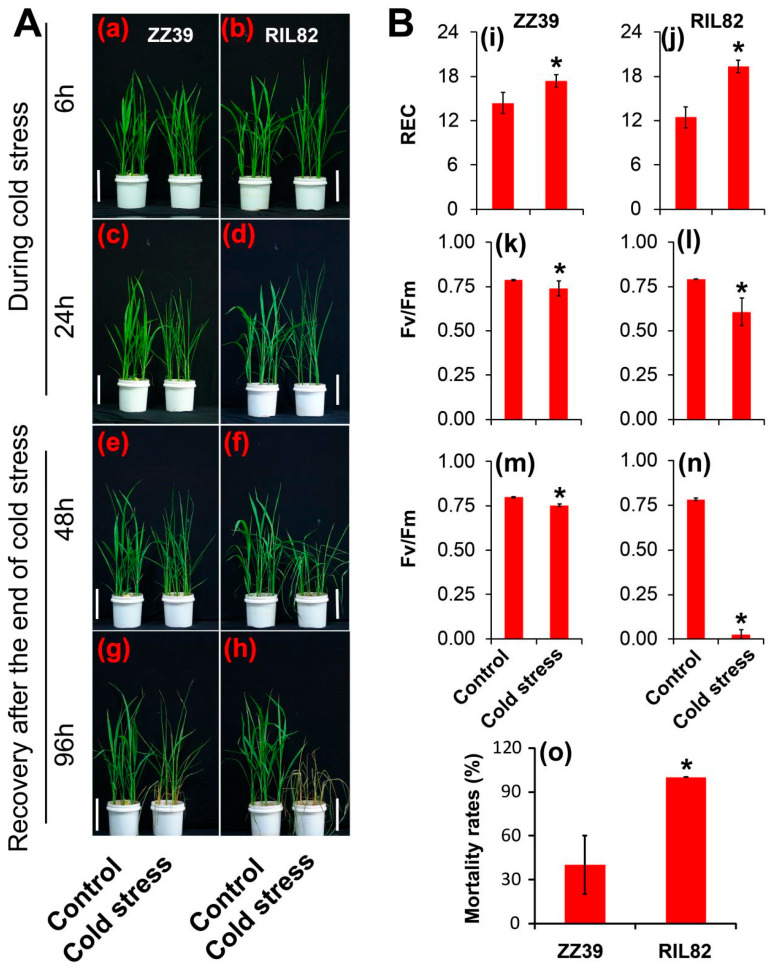
The response of rice plants to cold stress. (**A**), The morphology of rice plants under cold stress. (**a**,**b**), ZZ39 and RIL82 plants were subjected to cold stress for 6 h, respectively; (**c**,**d**), ZZ39 and RIL82 plants were subjected to cold stress for 24 h, respectively; (**e**,**f**), The recovery of ZZ39 and RIL82 plants for 48 h after the end of cold stress, respectively; (**g**,**h**), The recovery of ZZ39 and RIL82 plants for 96 h after the end of cold stress, respectively. (**B**), Changes in the REC, Fv/Fm and mortality rate of ZZ39 and RIL82 plants under cold stress. (**i**–**l**), The REC and Fv/Fm of ZZ39 and RIL82 plants under cold stress for 24 h; (**m**,**n**), Fv/Fm of ZZ39 and RIL82 plants recovered for 48 h after the end of cold stress; (**o**), Mortality rates of ZZ39 and RIL82 plants recovered for 96 h after the end of cold stress. REC, relative electrical conductance; Fv/Fm, maximum fluorescence quantum efficiency. The reference scales of (**a**–**h**), all represent 10 cm. Vertical bars denote standard deviations (*n* = 5). A *t*-test was adopted to compare the differences between the control and cold stressed within a cultivar. * denotes *p* < 0.05.

The two rice genotypes presented different morphologies after cold stress and recovery (Figure 1). No difference in leaf morphology was detected between the two genotypes under the control condition. Under cold stress, more damage was found on the leaves of RIL82 than on those of ZZ39 plants (Figure 1a–d). A significantly higher REC caused by cold stress was observed in RIL82 than in ZZ39 plants compared to their respective controls (Figure 1i,j). Cold stress decreased Fv/Fm in both genotypes, and notable reductions were found in RIL82 compared to those of ZZ39 plants regardless of the 24 h cold stress or 48 h recovery after the end of cold stress (Figure 1k–n). The mortality rate of RIL82 was significantly higher than that of ZZ39 plants after 96 h of recovery from the cold stress (Figure 1o).

### 3.2. Transcriptome Analysis of the Mechanism Underlying the Difference in Cold Tolerance between the Two Genotypes

**Figure 2:**
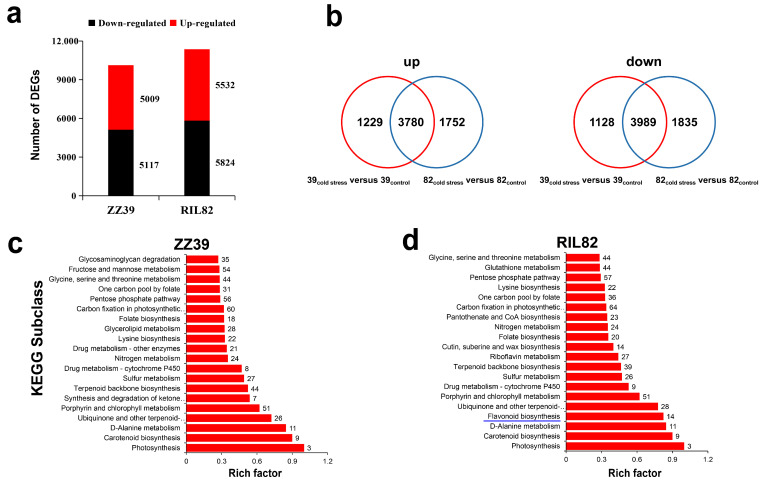
Transcriptome analysis in the two varieties under cold stress. (**a**), Number of DEGs in the ZZ39 and RIL82 genotypes after the cold treatment; (**b**), Venn diagram of upregulated DEGs (left) and Venn diagram of downregulated DEGs (right) in ZZ39 and RIL82; (**c**,**d**), Down KEGG enriched pathway (Top 20). The abscissa represents the Rich factor, and the ordinate represents the KEGG subclass. The blue line represents the flavonoid pathway played a key role in contributing to the difference in cold tolerance between the two genotypes.

**Figure 3:**
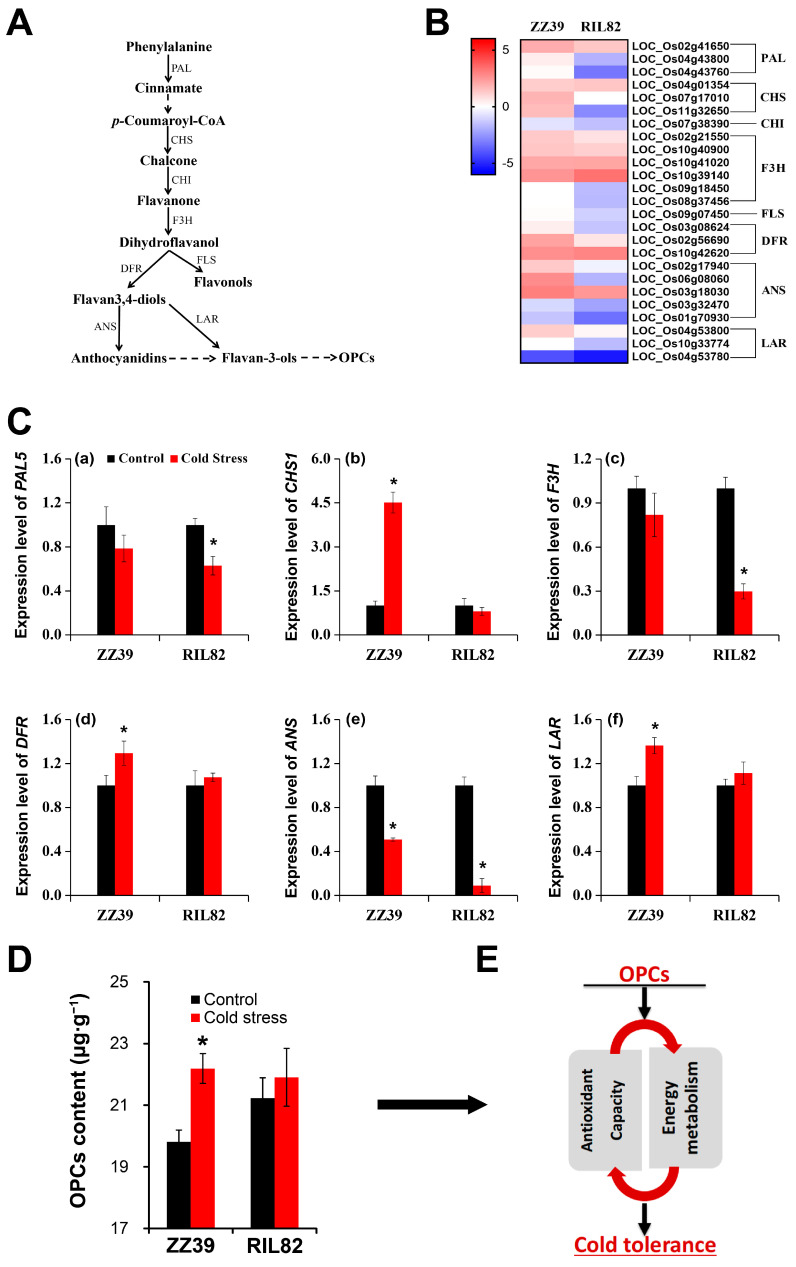
Transcriptome analysis on OPCs pathway in rice seedling plants under cold stress. (**A**), a schematic diagram on the synthesis OPCs; (**B**), heat map of enzyme genes involved in OPC synthesis (log_2_|FoldChange|); (**C**), Relative expression of enzyme genes involved in OPC synthesis; (**D**), OPC contents; (**E**), descriptive model of the relationships between OPCs, the antioxidant system, and energy homeostasis in plants under cold stress. OPCs, oligomeric proanthocyanidins. * denotes *p* < 0.05.

Transcriptome analysis was conducted to reveal the mechanism underlying the difference in cold tolerance between the two rice genotypes. Totals of 10,126 and 11,356 differentially expressed genes (DEGs) (fold-change > 2, *p* < 0.05) were found in ZZ39 and RIL82 plants, respectively (Figure 2a); 1752 upregulated and 1835 downregulated DEGs were detected in RIL82, while 1229 upregulated and 1128 downregulated DEGs were presented in ZZ39 plants, indicating to a certain extent, the two genotypes had distinct transcriptional differences under cold stress (Figure 2b). Gene ontology (GO) analysis showed that a great number of genes involving in biological process and metabolic process were disturbed by cold stress in both genotypes based on the total DEGs. However, the biosynthetic processes were more enriched in ZZ39 than in RIL82 plants, while the metabolic processes, particularly carbohydrate metabolism, were more enriched in ZZ39 than in RIL82 plants (Figure S1). In the case of cellular component enrichment analysis, many DEGs in ZZ39 were involved in cytoplasm and non-membrane-bounded organelle, while those of RIL82 were involved in membrane components (Figure S1). The Kyoto Encyclopedia of Genes and Genomes (KEGG) analysis showed that the flavonoid biosynthetic pathway was enriched in RIL82, while such a result was not presented in ZZ39 based on all of the DEGs (Figure S2). These results suggested that genes involved in biosynthetic process and metabolic process were differentially regulated by cold stress in the two rice genotypes. Interestingly, the flavonoid biosynthetic pathway was enriched in RIL82 only based on the downregulated DEGs (Figure 2c,d). This finding suggested that the flavonoid pathway played a key role in contributing to the difference in cold tolerance between the two genotypes. In addition, genes related to OPC biosynthesis in ZZ39 were upregulated under cold stress or no difference between the control and cold stress, while they were downregulated in RIL82 plants (Figure 3A,B). Similarly, the relative expressions of *PAL5*, *F3H* and *ANS* were decreased by cold stress, in which a smaller decrease was presented in ZZ39 than RIL82 plants (Figure 3C(a,c,e)). In contrast, notable increase in the relative expressions of *CHS1*, *DFR* and *LAR* genes were presented in ZZ39 plants, while no significant difference was found in RIL82 (Figure 3C(b,d,f)). Accordingly, a notable increase in OPC contents was detected in ZZ39 plants under cold stress compared to the control, while in RIL82 no difference was found between the cold stressed and control treatments (Figure 3D). Additionally, the genes related to the antioxidant capacity and energy metabolism were also involved in this process (Figure S3). We selected five genes for qRT-PCR, and similar expression patterns were found between the qRT-PCR and RNA-seq analyses of both genotypes (Figure S4). Therefore, we inferred that OPCs, antioxidant capacity and energy metabolism might be involved in contributing to the cold tolerance between two rice genotypes (Figure 3E).

### 3.3. Effect of OPCs on the Morphology, REC, H_2_O_2_, and Fv/Fm of Rice Leaves under Cold Stress

**Figure 4:**
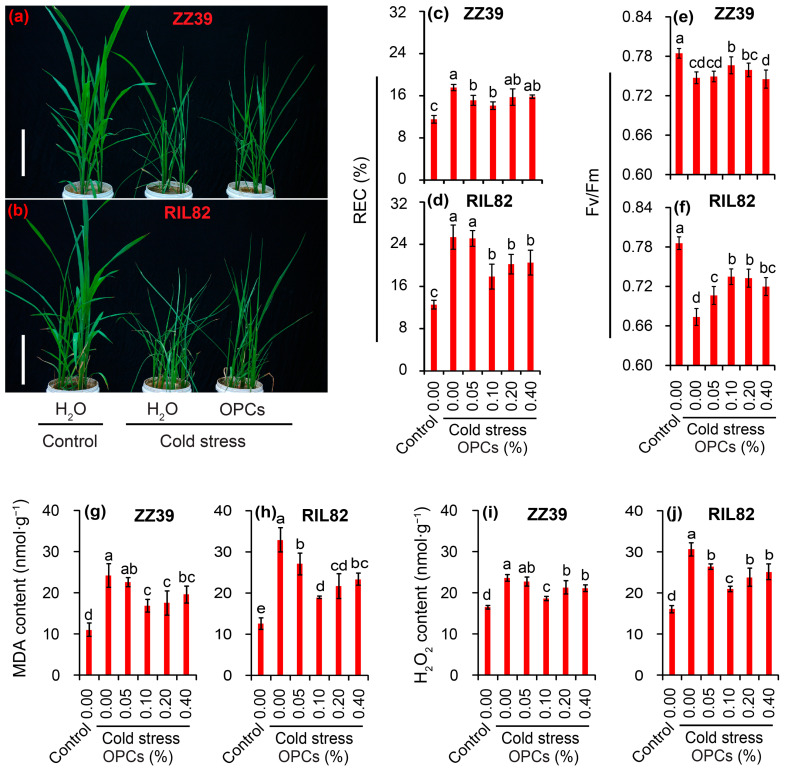
Effects of OPCs on rice plants under cold stress. (**a**,**b**), The leaf morphology of rice plants; (**c**,**d**), REC; (**e**,**f**), Fv/Fm; (**g**,**h**), MDA; (**i**,**j**), H_2_O_2_. OPCs, oligomeric proanthocyanidins; REC, relative electric conductance; Fv/Fm, maximum fluorescence quantum of PSII; MDA, malondialdehyde; H_2_O_2_, hydrogen peroxide. The reference scales of (**a**,**b**) represent 10 cm. Vertical bars denote standard deviations (*n* = 3). Different letters indicate significant differences among the control and cold stress treatments within one genotype by two-way analysis of variance (temperature and treatment) (*p* < 0.05).

Based on the above results, we speculate that OPCs might be involved in affecting cold tolerance between the two rice genotypes. To confirm this hypothesis, different OPC concentrations were sprayed onto the two rice genotypes under cold stress, and the plant morphology, REC and Fv/Fm as well as contents of H_2_O_2_ and MDA were investigated. The results indicated that the OPCs could improve the plant morphology of the two rice genotypes under cold stress (Figure 4a,b). The REC, as well as the MDA and H_2_O_2_ contents, increased significantly under cold stress, whereas these enhancements were reduced by the OPCs (Figure 4c,d,g,i). The lowest REC, MDA, and H_2_O_2_ values under cold stress were observed in plants treated with 0.1% OPCs, which was significantly lower than those of plants treated with H_2_O. The Fv/Fm value decreased significantly in response to the cold stress in both genotypes; a remarkable increase in Fv/Fm was observed in rice plants treated with 0.1% OPCs compared to those plants treated with H_2_O under cold stress, particularly in the RIL82 (Figure 4e,f). According to these results, rice seedling plants treated with 0.1% OPCs could obviously increase the cold tolerance through reducing REC as well as contents of H_2_O_2_ and MDA.

### 3.4. Effects of OPCs on Antioxidant Enzyme Activities of Leaves under Cold Stress

**Figure 5:**
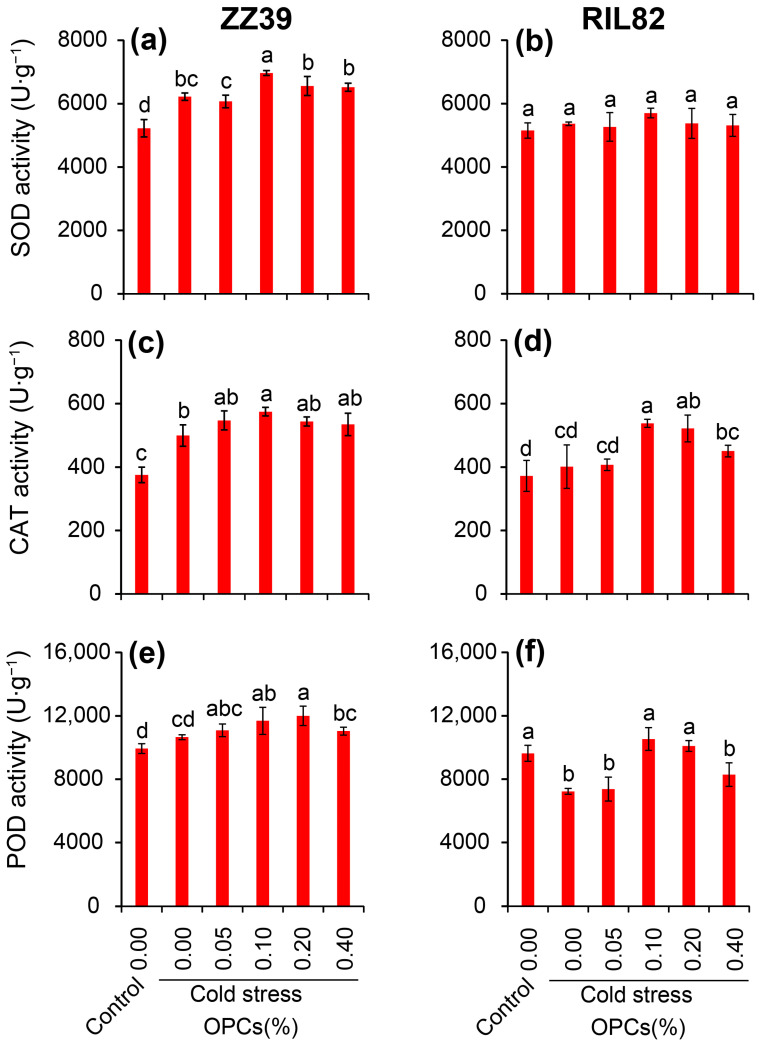
Effect of OPCs on antioxidant enzyme activities in rice leaves under cold stress. (**a**,**b**), SOD activity; (**c**,**d**), CAT activity; (**e**,**f**), POD activity. OPCs, oligomeric proanthocyanidins; SOD, superoxide dismutase; CAT, catalase; POD, peroxidase. Vertical bars denote standard deviations (*n* = 3). Different letters indicate significant differences among the control and cold stress treatments within a genotype by two-way analysis of variance (temperature and treatment) (*p* < 0.05).

The activities of SOD, POD, and CAT were determined to reveal the functions of OPCs in the antioxidant capacity in rice plants under cold stress. The SOD activity of ZZ39 increased significantly under cold stress; in this stress conditions a remarkable increase in SOD activity were observed in the plants treated with 0.1% OPCs compared with that of H_2_O treatment (Figure 5a). Such results were not found in the RIL82 plants under cold stress, in which no obvious difference in SOD activity were detected among all the treatments (Figure 5b). Cold stress significantly increased CAT activity in the leaves of ZZ39 but did not affect the leaves of RIL82 plants (Figure 5c,d). CAT activity was induced by OPCs in the two genotypes under cold stress, and the highest values were found in the 0.1% OPC treatment, which was significantly higher than that of the H_2_O treatment. No significant difference in POD activity was detected between the control and cold stressed ZZ39 plants, while a significant reduction was observed in RIL82 caused by cold stress compared to the control (Figure 5e,f). Similarly, POD activity increased in response to the OPCs under cold stress in both genotypes; the POD activities of the 0.1% OPC and 0.2% OPC treatments were significantly higher than those of the H_2_O treatments. In sum, the antioxidant capacity including SOD, POD, and CAT activity were enhanced by 0.1–0.2% OPCs in rice plants under cold stress.

### 3.5. Effects of OPCs on NSC and Energy Metabolism of Rice under Cold Stress

**Figure 6:**
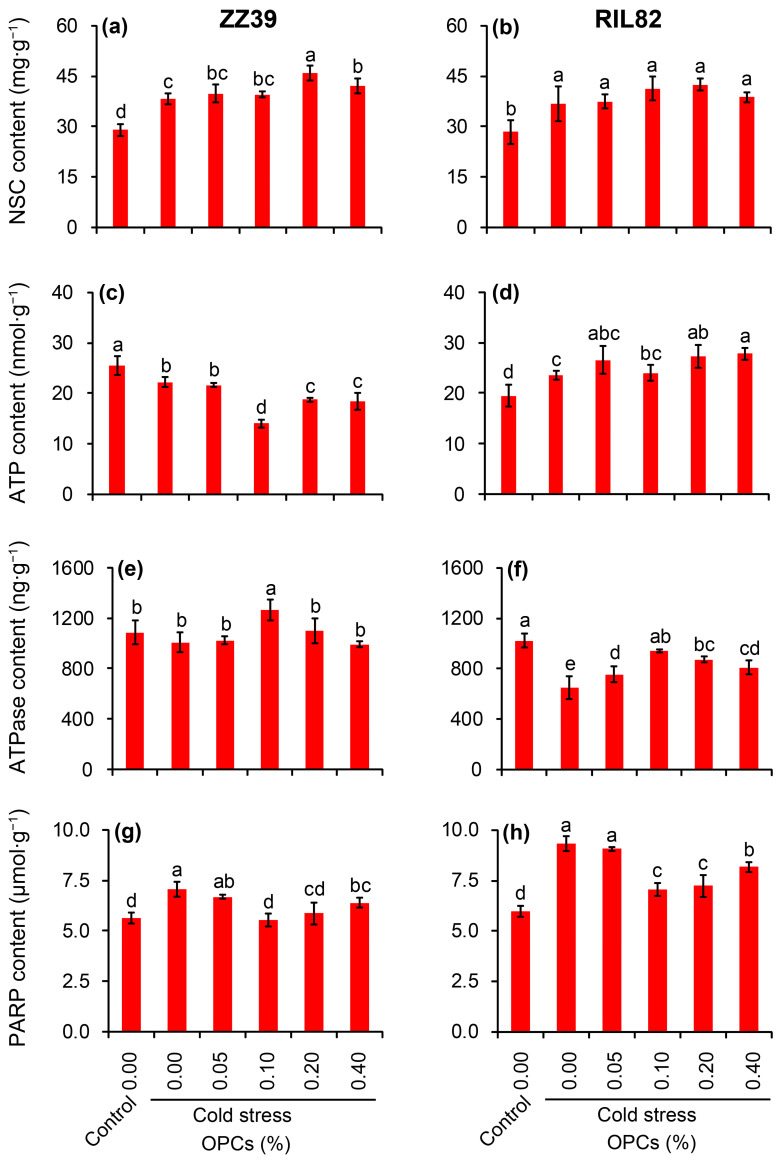
Effect of OPCs on NSC content, ATP metabolism, and PARP content of rice leaves under cold stress. (**a**,**b**), NSC content; (**c**,**d**), ATP content; (**e**,**f**), ATPase content; (**g**,**h**), PARP content. OPCs, oligomeric proanthocyanidins; NSC, non-structural carbohydrates; PARP, poly (ADP-ribose) polymerase. Vertical bars denote standard deviations (*n* = 3). Different letters indicate significant differences among the control and cold stress treatments within a genotype by two-way analysis of variance (temperature and treatment) (*p* < 0.05).

Sugars and energy are important factors involved in OPCs affecting antioxidant capacity in rice seedlings under cold stress, and thus the contents of NSC, ATP, ATPase and PARP were determined. The NSC contents in the rice plants increased in response to the cold stress, but no significant difference was observed between the genotypes (Figure 6a,b). Under cold stress, the 0.2% and 0.4% OPC treatments had greater effects on the NSC content of ZZ39, both of which were significantly higher than the H_2_O treatment. However, no significant difference was observed among all of the cold stress treatments in RIL82 plants. The effect of cold stress on ATP content differed between the genotypes (Figure 6c,d). A notable reduction in ATP content was found in ZZ39 plants under cold stress compared to the control, while ATP increased significantly in RIL82 under cold stress. The ATP content of ZZ39 plants under cold stress treated with 0.1, 0.2, or 0.4% OPCs was significantly lower than that of the H_2_O treatment, in which the lowest value was found in plants treated with 0.1% OPCs. In contrast, the ATP contents of the 0.2 and 0.4% OPC treatments were significantly higher than that of the H_2_O treatment in RIL82 plants; no difference in ATP content was detected between the 0.1% OPC and H_2_O treatments. Cold stress had little effect on ATPase activity in ZZ39 compared to the control, whereas a significant decrease in ATPase activity was found in RIL82 plants (Figure 6e,f). The ATPase activity of the 0.1% OPC treatment was significantly higher in ZZ39 plants under cold stress than in the other treatments, and no differences were observed among the other treatments. OPCs alleviated the inhibited ATPase activity caused by cold stress in RIL82 plants; the ATPase activity of the OPC treatments was significantly higher than that of the H_2_O treatments under cold stress, in which the highest value was in the 0.1% OPC treatment. PARP can be activated by cold stress, and a greater increase in PARP activity was found in RIL82 than in ZZ39 plants under cold stress compared to their respective controls (Figure 6g,h). The PARP activity of the 0.1% and 0.2% OPC treatments in ZZ39 was lower than that of the H_2_O treatment, and significant decreases in PARP activity were found in the 0.1, 0.2, and 0.4% OPC treatments compared to the H_2_O treatments in RIL82 plants. It was considered that, 0.1% OPCs could improve the energy status in rice seedling plants mainly ascribing to its function in enhancing the ATPase content but inhibiting the PARP content.

### 3.6. Effect of the OPCs + 3ab Combination on Fv/Fm, H_2_O_2_, and Energy Metabolism under Cold Stress

**Figure 7:**
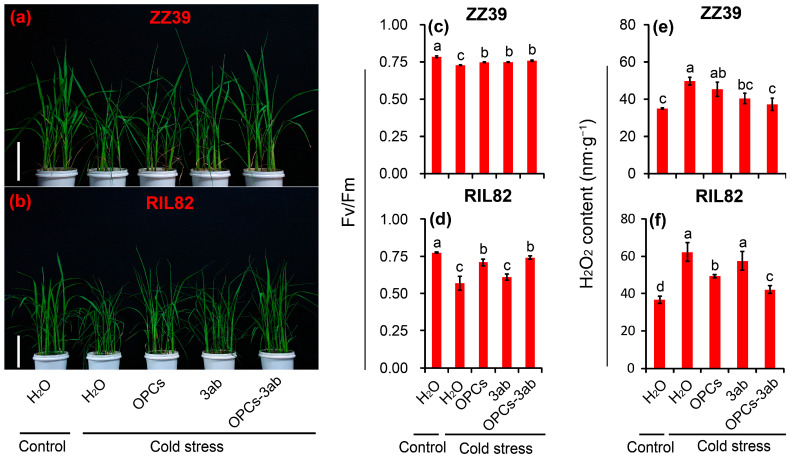
Effect of OPCs and 3-ab on leaf morphology, H_2_O_2_ content, and Fv/Fm in rice leaves under cold stress. (**a**,**b**), leaf morphology; (**c**,**d**), Fv/Fm; (**e**,**f**), H_2_O_2_ content. OPCs, oligomeric proanthocyanidins; 3-ab, 3-aminobenzamide; Fv/Fm, Maximum fluorescence quantum of PSII; H_2_O_2_, hydrogen peroxide. The reference scales of (**a**,**b**) represent 10 cm. Vertical bars denote standard deviations (*n* = 3). Different letters indicate significant differences among the control and cold stress treatments within a genotype by two-way analysis of variance (temperature and treatment) (*p* < 0.05).

Our results indicate that OPCs might enhance cold tolerance in rice seedling plants by improving antioxidant capacity and energy status. To confirm this hypothesis, the OPCs and 3ab alone or combination were sprayed onto rice seedling plants under cold stress. The 3ab, a PARP inhibitor, can suppress the PARP activity to reduce the consumption of NAD^+^, that increases the energy production efficiency and improve energy status in plants. Thus, the Fv/Fm, and contents of H_2_O_2_, ATP, ATPase, and PARP were determined to investigate the energy status in seedling plants under cold stress. OPCs, 3ab, and OPCs + 3ab alleviated the cold damage compared to the plants treated with H_2_O in the two genotypes, particularly the OPCs + 3ab treatment (Figure 7a,b). No difference in Fv/Fm was observed among the OPCs, 3-ab, or OPCs + 3ab treatments in ZZ39 plants under cold stress, whereas they were significantly higher than the H_2_O treatment. The highest Fv/Fm value in RIL82 plants was observed in the OPCs and OPCs + 3ab treatments, which were significantly higher than those of the H_2_O and 3ab treatments; no difference in Fv/Fm was showed between the H_2_O and 3ab treatments in RIL82 plants under cold stress (Figure 7c,d). H_2_O_2_ contents increased significantly in the two genotypes under cold stress compared to the control (Figure 7e,f). Among these treatments, the lowest values were found in the OPCs + 3ab treatment, followed by the 3ab treatment in ZZ39 plants, which was significantly lower than the H_2_O treatment under cold stress. In RIL82 plants, the lowest H_2_O_2_ content was found in the OPCs + 3ab treatment, followed by the OPC treatments, both of which were significantly lower than the H_2_O treatment under cold stress. All these indicated that synergistic effect was existed in OPCs and 3ab, since a notable increase in cold tolerance was presented in OPCs + 3ab treatment compared with the other treatments.

**Figure 8:**
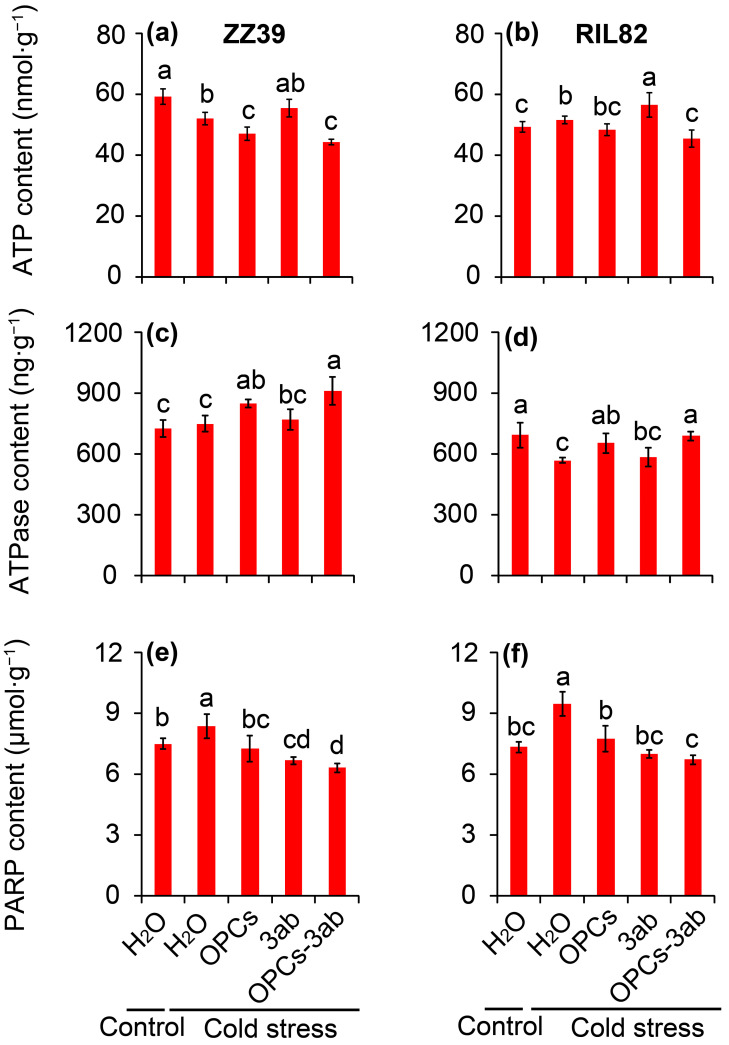
Effect of OPCs and 3ab on contents of ATP, ATPase, and PARP in rice leaves under cold stress. (**a**,**b**), ATP content; (**c**,**d**), ATPase content; (**e**,**f**), PARP content. OPCs, oligomeric proanthocyanidins; 3ab, 3-aminobenzamide; PARP, poly (ADP-ribose) polymerase. Vertical bars denote standard deviations (*n* = 3). Different letters indicate significant differences among the control and cold stress treatments within a genotype by two-way analysis of variance (temperature and treatment) (*p* < 0.05).

Regarding the energy status, the highest ATP content under cold stress was detected in the 3ab treatment, followed by the H_2_O treatment, while the lowest values were observed in the OPCs + 3ab treatments of the two genotypes (Figure 8a,b). In contrast, the highest ATPase activity was observed in plants treated with OPCs + 3ab, followed by the OPCs treatments, both of which were significantly higher than that of the H_2_O treatments in the two genotypes under cold stress (Figure 8c,d). PARP activity decreased significantly compared to the H_2_O treatment when plants were sprayed with the OPCs, 3-ab, and OPCs + 3ab treatments under cold stress in the two genotypes; the lowest values were found in the OPCs + 3ab treatment (Figure 8e,f). Thus, these results suggested that OPCs could synergy with 3ab to increase cold tolerance in rice seedling plants by improving energy status.

## 4. Discussion

Cold stress damaged the rice plants in both genotypes. The mortality rates of the ZZ39 and RIL82 plants under cold stress were 40% and 100% respectively, suggesting that the ZZ39 had higher cold tolerance than that of the RIL82 plants (Figure 1). This finding was consistent with previous results reported by Yu et al. [39], who reported that glutathione (GSH) was the main factor contributing to the difference in cold tolerance between these two genotypes. According to the transcriptome analysis, genes related to GSH were induced and were involved in regulating the cold response in ZZ39 and RIL82 plants (Figure 2c,d). However, the genes related to flavonol in RIL82 plants decreased in response to the cold stress, whereas no significant difference was observed between the control and cold stressed in ZZ39 plants (Figure 3A,B), suggesting that flavonols also notably affected cold response in these two genotypes [65,66,67].

As the final product of the flavonol pathway in plants, OPCs are abundant but complex class of polyphenols found in foods and botanicals, which has been largely utilized in animals and humans to treat disease because of their strong antioxidant properties [30]. Several researches have reported that the OPCs could affect the resistance to abiotic stress [68,69,70,71]. However, the role of OPCs in rice plants under abiotic stress has rarely been documented [72], particularly under cold stress. The present results revealed a significant increase in OPCs contents in ZZ39 plants under cold stress compared to the control, while no difference in OPCs contents was detected in RIL82 plants between the control and cold stressed (Figure 3D). Importantly, exogenous OPCs alleviate cold damage in rice plants, particularly in the cold susceptible cultivar RIL82 (Figure 4), indicating that OPCs also conferred cold tolerance to the rice seedlings, which was consistent with the result conducted with apple [34].

As reported, many genes and transcription factors are involved in the OPCs synthesis and stress response in plants [31,73,74,75,76]. Similarly, such results were also found in rice seedling plants according to the transcriptome analysis (Figure 2 and Figure 3). Several genes including *PAL5*, *F3H*, *ANH*, *CHS1*, *DFR* and *LAR* played important roles in the difference accumulation of OPCs and cold tolerance between these two rice genotype (Figure 3C). As well-known, many PAs are oligomers of the catechin and epicatechin flavonoid compounds, and key steps in the synthesis of these two building blocks are catalyzed by leucoanthocyanidin reductase (LAR) and anthocyanidin reductase (ANR), respectively. Therefore, among these genes, the *ANR* and *LAR* are two key genes responsible for the synthesis of OPCs [74], both of which confer abiotic or biotic resistance to plants. However, the *ANR* were largely documented in affecting abiotic or biotic stress compared with *LAR* [31,77], in particular under cold stress; The R2R3-MYB transcription factor *MdMYB23* could interact with the promoter of ANR to enhance the cold tolerance and proanthocyanidins accumulation in apple [34]. Up to now, the effect of *LAR* on rice plants under cold stress has not been reported. However, the expression levels of *LAR* were increased in plants under cold stress compared with those of controls in two rice genotypes, while large decreases were presented in *ANS* (Figure 3). This suggested that *LAR* could also enhance cold tolerance in rice seedling plants by accumulating OPCs, whereas more researches are required to reveal the underlying mechanism.

Large increases in the activities of SOD, POD, and CAT, as well as large decreases in MDA and H_2_O_2_ contents, were found in the plants treated with the OPCs under cold stress compared to those plants treated with H_2_O (Figure 4 and Figure 5). Thus, the anti-stress function of OPCs in plants may be because of their strong antioxidant capacity [31,78,79]. Antioxidant capacity in plants is very energy costly; thus, stress tolerance may become impaired under low energy conditions [37]. This could explain why plants with higher stress tolerance always compromise biomass, yield, or quality, as most of the energy is allocated to maintain respiration, rather than growth [38,80]. Interestingly, the ATP content in the rice plants treated with OPCs was significantly lower than that of plants treated with H_2_O under cold stress in ZZ39 seedling plants (Figure 6c,d). This result suggested that OPCs enhanced cold tolerance by increasing energy utilization efficiency, which was determined by ATPase activity. It has been reported that the ATP hydrolysis blocked by the lower ATPase activity has been considered as the main factor resulting in more damage to RIL82 plants under cold stress [39]. Furthermore, OPCs could pose significant effects on the fermentation via glucose transport, the energy and redox homeostasis as well as the activities of rate-limiting enzymes in glycolysis [81]. Thus, OPCs increased energy use efficiency by regulating ATPase activity, which is responsible for the strong antioxidant capacity in plants under cold stress. This finding was confirmed by the result that cold tolerance was enhanced in rice plants treated with OPCs + 3ab compared to the other treatments in the two genotypes, but no difference in cold tolerance was found between the H_2_O and 3ab treatments of RIL82 plants; a significantly higher ATP content was found in the 3ab than the H_2_O treatment under cold stress (Figure 7 and Figure 8).

The ATPases, including the phosphorylated intermediate-type (P-type) and vacuolar-type (V-type) H^+^-ATPases, are important ATP-driven proton pumps that generate membrane potential and provide proton motive force for secondary active transport [71]. The expression and activity of both P- and V-type H^+^-ATPases are highly regulated by hormones and environmental cues, which are involved in plant growth and stress adaptation [71]. Indeed, the H^+^-ATPase is required for proanthocyanidins synthesis in the Arabidopsis thaliana seeds or Gossypium hirsutum [28,49,50,82]. Interestingly, some of the flavonoids, including Myricetin, quercetin (C) and gossypin were found to be inhibitors of K^(+)^-ATPase, which was competitive with respect to ATP [83,84,85,86]. However, the OPCs could increase the activity of ATPase and fermentation efficiency [81]. Moreover, OPCs significantly reduced the concentration of free Ca^2+^ and elevated Ca^2+^-ATPase activity in sciatic nerves of rats [87]. This finding was consistent with the present results that higher ATPase activity were found in plants treated with OPCs than those plants treated with H_2_O (Figure 6e,f). This novel function of OPCs may be related to their ability to reduce excess ROS (Figure 4i,j), which always inhibits ATPase in plants under abiotic stress [88,89,90,91].

**Figure 9:**
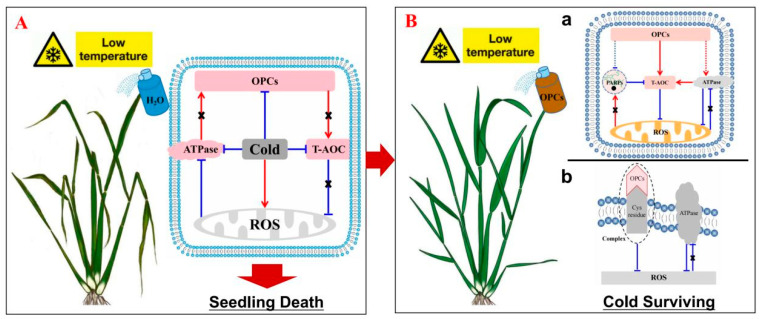
Descriptive model of the function of OPCs in conferring cold tolerance in rice. (**A**), Cold stress significantly inhibits OPC synthesis, impairs T-AOC and ATPase activity, resulting in excess ROS accumulation, leading to seedling death. (**B**) (a), Exogenous OPCs enhance T-AOC and reduce the excess ROS caused by cold stress. This alleviates the activation of PARP, inhibition on ATPase activity of rice under cold stress, and thus provides sufficient available energy for the formation of antioxidants. (**B**) (b), OPCs combine with Cys residues to form a complex at the plasma membrane to scavenge intracellular ROS to stabilize ATPase activity under cold stress [92]. Taken together, OPCs could improve energy status to enhance cold tolerance in rice plants. OPCs, oligomeric proanthocyanidins; PARP, poly (ADP-ribose) polymerase; T-AOC, total antioxidant capacity; ROS, reactive oxygen species; Cys residue, cysteine residue.

Indeed, the ATPase including Na^+^/K^+^, Ca^2+^, and H^+^ pumping P-type ATPases, and V-ATPase are subject to redox regulation in mammals, yeast and plants [88]. Oxidative inhibition of the ATPase is ascribed to disulfide-bond formation between conserved cysteine residues at the catalytic site of subunit A, which can be induced by the reactive oxygen species [89]. The Cys-327 functions as a protective residue in the plasma membrane H^+^-ATPase, and other P-type ATPases [91]. It has been reported that the oligomeric proanthocyanidin-L-cysteine complexes presented higher bioavailability and antioxidant capacity and enhanced survival time in the animal test groups [92]. Thus, OPCs was inferred to be directly combined with cysteine residues to stabilize ATPase caused by cold stress in a ROS independent pathway, which has not been documented previously (Figure 9). However, increased H^+^-ATPase could improve oxidative stress in Candida glabrata, Tamarix hispida Willd and tea plants [93,94,95].

The function of OPCs in conferring strong antioxidant capacity in rice plants to maintain ROS homeostasis under cold stress could reduce energy consumption (Figure 9). Knowingly, PARP uses NAD^+^ to produce the post-translationally modified PAR that attaches to PARP itself or other target proteins [96]. This process costs a large amount of energy. PARP is activated by abiotic stressors, such as hot and cold [37,39,97,98]. A significant increase in PARP activity was detected in plants under cold stress, in which the greater increase was observed in RIL82 than in ZZ39 plants (Figure 6g,h). Interestingly, OPCs inactivated PARP, and conferred cold tolerance in rice plants, particularly in the cold-susceptible RIL82 cultivar. This finding suggests that OPCs could also reduce energy consumption in plants under cold stress (Figure 9). This function of OPCs may be related to their ability to scavenge excess ROS, which activates PARP under cold stress [39,45].

## 5. Conclusions

Cold stress caused more damage to RIL82 than ZZ39 plants. Transcriptome analysis indicated that OPCs were involved in affecting the cold tolerance between the two genotypes. This hypothesis was confirmed by that data that 0.1% OPCs significantly enhanced cold tolerance in plants by improving antioxidant capacity and reducing excess ROS. ATP content was lower in plants under cold stress treated with OPCs than that in plants treated with H_2_O, while higher ATPase activity was found in plants treated with OPCs than plants treated with H_2_O. This result indicates that OPCs enhanced energy use efficiency to provide sufficient energy for antioxidant capacity under cold stress. Further, OPCs inactivated PARP to reduce energy consumption in plants under cold stress. Taken together, OPCs enhanced ATPase activity and inhibited PARP activity to improve energy status and confer cold tolerance in plants by maintaining the oxidant balance.

## Data Availability

All data are contained within the article.

## Supplementary Materials

The following supporting information can be downloaded at: https://www.mdpi.com/article/10.3390/antiox12010079/s1, Figure S1: Gene Ontology (GO) analysis (TOP30); Figure S2: Kyoto Encyclopedia of Genes and Genomes (KEGG) analysis (TOP20); Figure S3: Heat map of genes associated with antioxidant capacity and energy metabolism; Figure S4: Histogram of five selected genes with changes measured by RNA-seq and qRT-PCR (ZZ39cold stress/ZZ39control, RIL82cold stress/RIL82control).; Table S1: Primer sequences for the genes used in qRT-PCR.
