# Supplementary material for: Oligomeric Proanthocyanidins Confer Cold Tolerance in Rice through Maintaining Energy Homeostasis
Source: Antioxidants (Basel). 2022 Dec 29;12(1):79. doi: 10.3390/antiox12010079 (PMC9854629; doi:10.3390/antiox12010079)
Supplement: Supplementary file 1 [file antioxidants-12-00079-s001.zip › Table S1.pdf]

Table S1. Primers sequences of the genes used in qRT-PCR

Gene name	Gene ID	Forward primer	Reverse primer
OsA2	LOC_Os07g09340	GCCGCACATCTTGTCGATTC	CCCTGTGTTGGATGGGGTAC
ATPase activity	LOC_Os10g36530	GAAGGAGCTGCTTTGGGGAT	CATGCACGCGTTCTCAAACA
ATPase activity	LOC_Os01g50080	TGTGTGCTTTAGCTACCCCG	ACCACTTCCACTTTGCCCAA
ATPase activity	LOC_Os07g32560	CTTCTACGTCGGCAACACCT	GGTGAGGTAGTTGAGCGTGT
PAL5	LOC_Os04g43760	TCGATGGCAAGAAGGTGGAC	TTCACGATGGCGAGACCTTC
F3H	LOC_Os08g37456	TGGGGCTTCTTCATGGTGAC	TCTGAACAGCTCCTTGCAGG
FLS	LOC_Os09g07450	GGCAATCCCCTTGATCGACA	CCTCCCTACATGCATCGTCC
ANS	LOC_Os01g70930	GACGCTGGAGAAGTACTCGG	AAGTTCATCCTCACCGCCTG
CHS1	LOC_Os11g32650	GCAGAAGGCGATCAAGGAGT	ATCATGAGGCGGTTCACGTT
DFR	LOC_Os02g56690	CTGGAGCGACCCCAAATTCT	CGTGGTTGATGCTGGTGTTG
LAR	LOC_Os04g53800	TCTCTCGGAGAAGGAAGCGA	ACAGCAAGGAGAGGACGTTG
