# Supplementary figures and images for: Oligomeric Proanthocyanidins Confer Cold Tolerance in Rice through Maintaining Energy Homeostasis
Source: Antioxidants (Basel). 2022 Dec 29;12(1):79. doi: 10.3390/antiox12010079 (PMC9854629; doi:10.3390/antiox12010079)

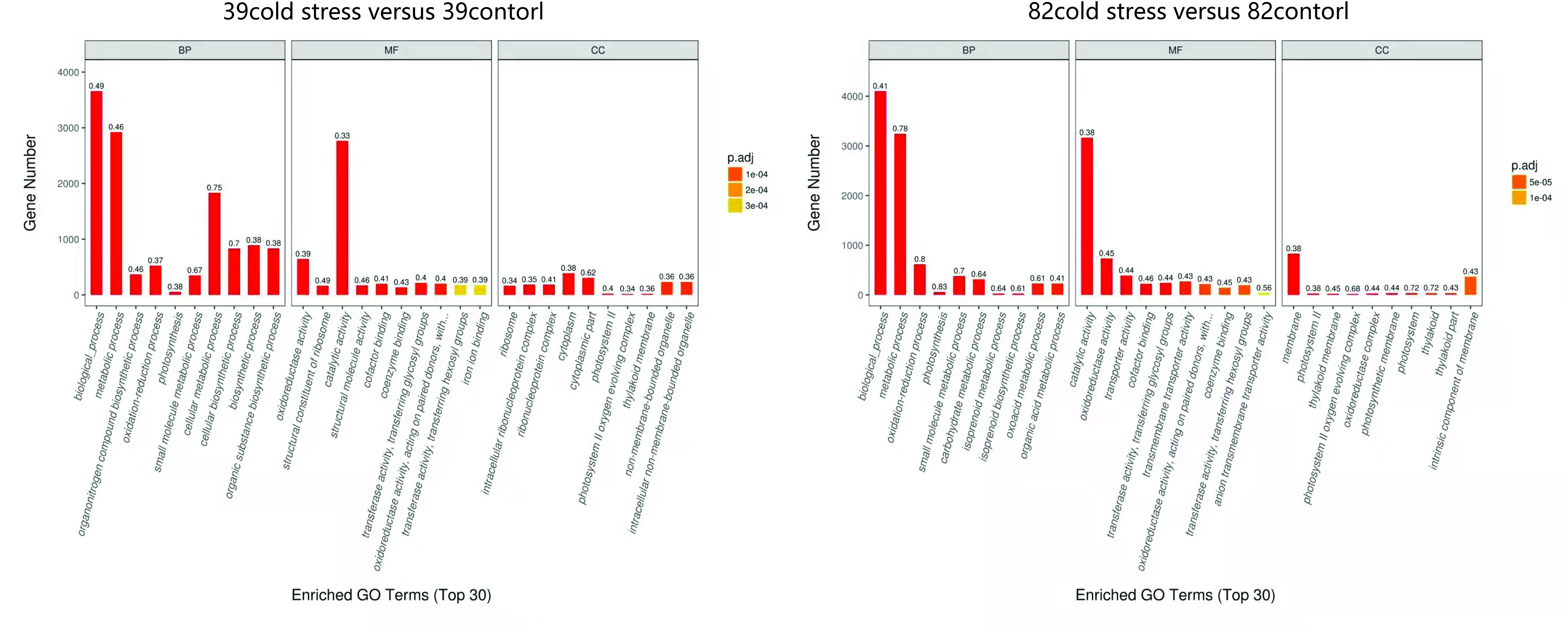

Supplement: Supplementary file 1 [file antioxidants-12-00079-s001.zip › Figure S1.jpg]

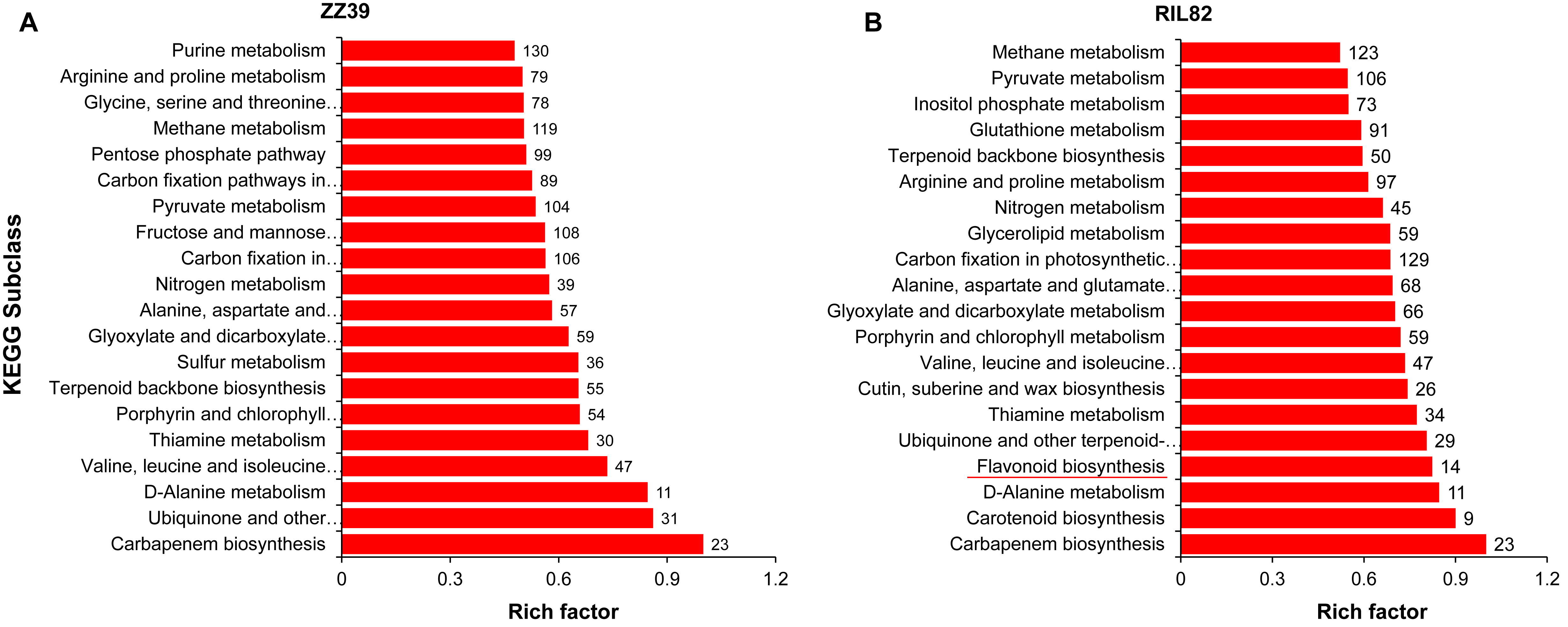

Supplement: Supplementary file 1 [file antioxidants-12-00079-s001.zip › Figure S2.jpg]

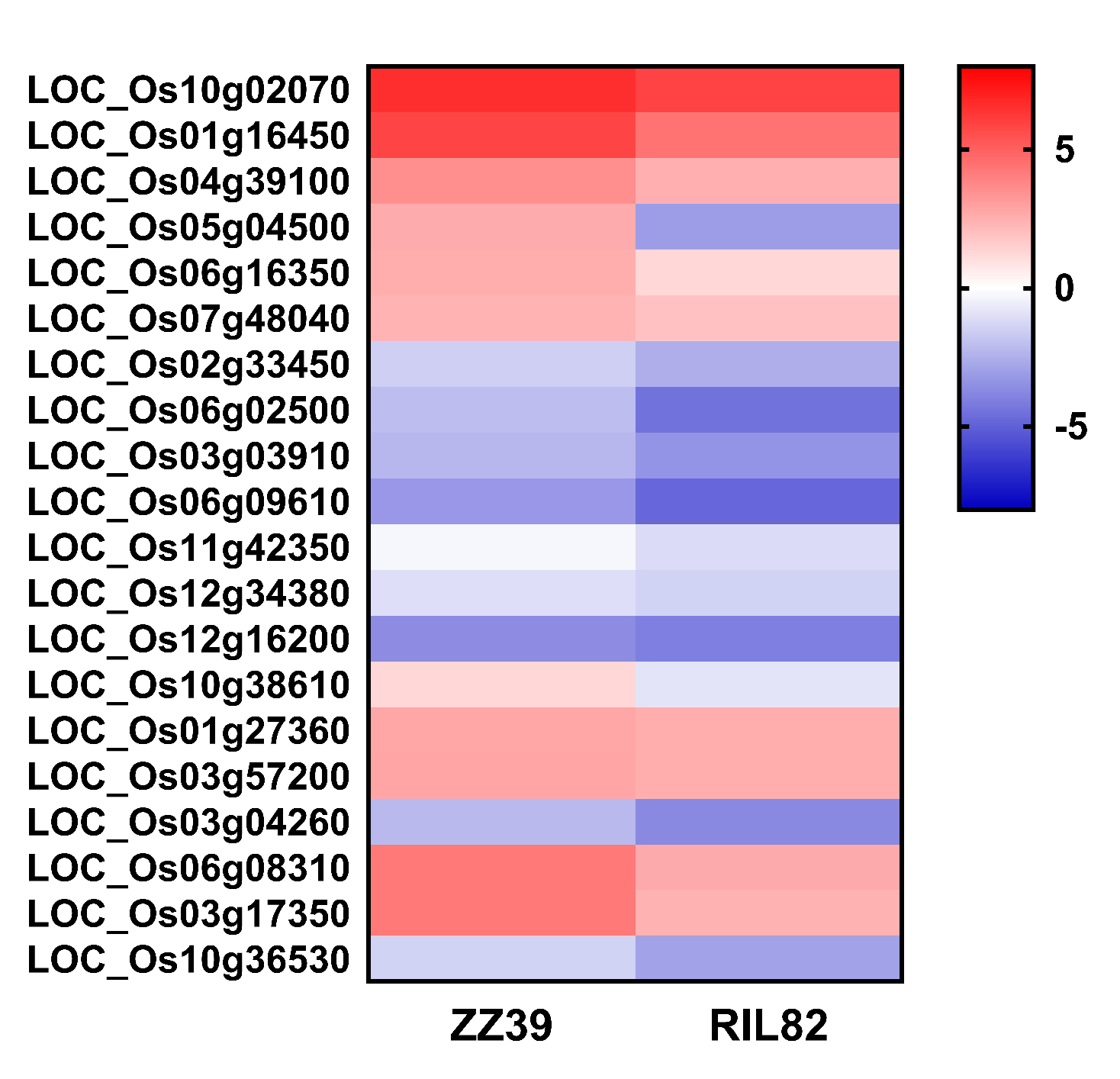

Supplement: Supplementary file 1 [file antioxidants-12-00079-s001.zip › Figure S3.jpg]

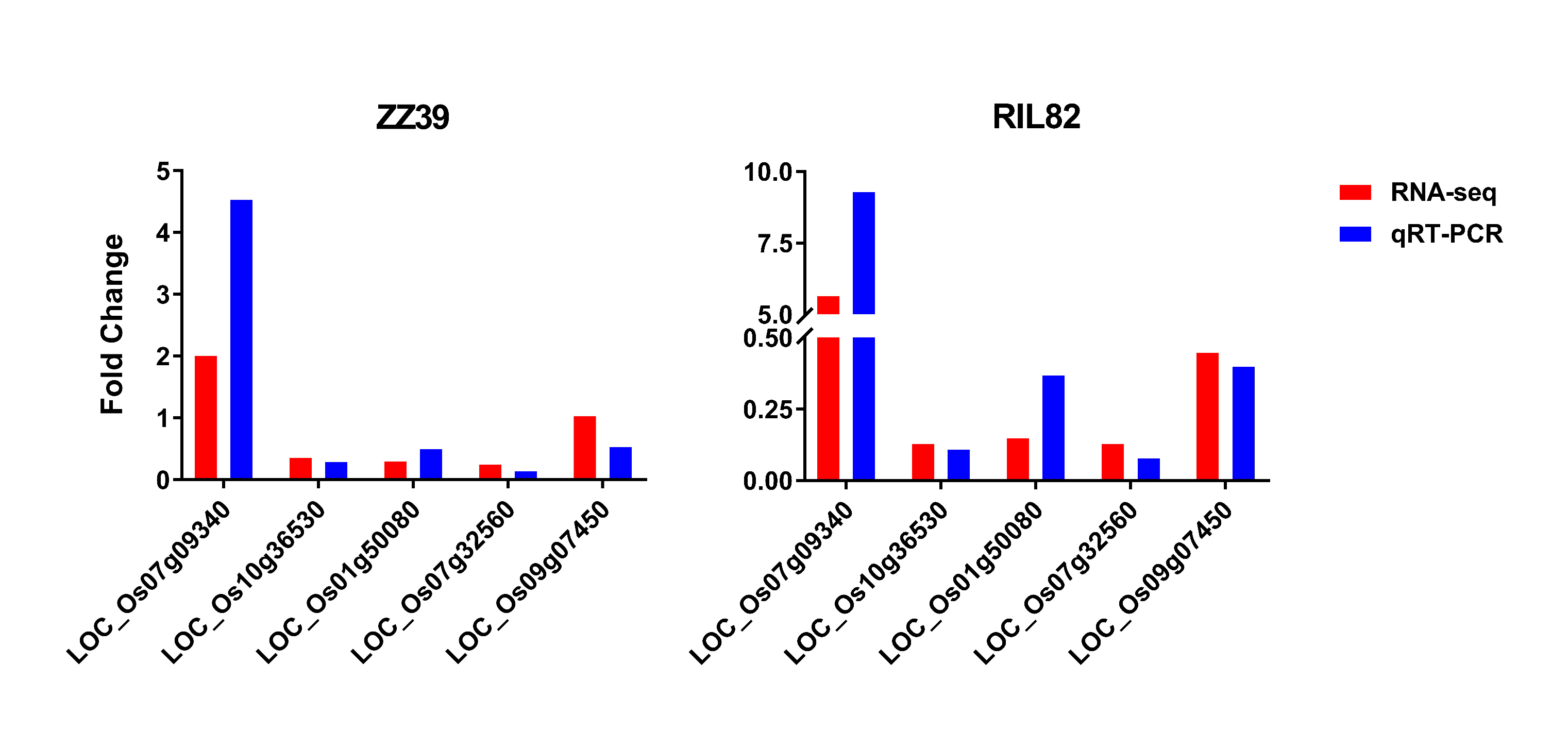

Supplement: Supplementary file 1 [file antioxidants-12-00079-s001.zip › Figure S4.jpg]
